# Posterior Ankle Pain in a Ballet Dancer: Dynamic Ultrasonography of Simultaneous Onset of Subtalar Joint Arthritis and Os Trigonum Syndrome

**DOI:** 10.7759/cureus.92241

**Published:** 2025-09-13

**Authors:** Hidenori Futamura, Masashi Kawabata, Hiroki Nakagawa, Ryo Futamura, Katsumasa Sugimoto

**Affiliations:** 1 Department of Rehabilitation, Nagoya Sports Clinic, Nagoya, JPN; 2 Department of Rehabilitation, Kitasato University School of Allied Health Sciences, Sagamihara, JPN; 3 Department of Orthopaedics, Nagoya Sports Clinic, Nagoya, JPN

**Keywords:** ballet, dynamic ultrasonography, os trigonum, posterior ankle impingement, subtalar joint arthritis

## Abstract

Posterior ankle pain during plantar flexion is frequently observed in ballet dancers, with os trigonum syndrome being one potential cause. However, concomitant subtalar joint pathology has been rarely reported. We herein present the case of a teenage female ballet dancer who presented with persistent posterior ankle pain during relevé movements, initially diagnosed with os trigonum syndrome based on computed tomography (CT) and ultrasonography, which also revealed abnormalities around the flexor hallucis longus (FHL) tendon. Despite a 15-day rest period and targeted physiotherapy focused on improving the FHL tendon gliding, her symptoms persisted. Re-evaluation revealed localized tenderness over the posterior talocalcaneal facet of the subtalar joint and pain reproduction during maximal ankle plantar flexion combined with forced subtalar pronation. Dynamic ultrasonography revealed a hypoechoic intra-articular lesion migrating during joint space narrowing, thereby reproducing the symptoms. Magnetic resonance imaging demonstrated joint effusion, and CT revealed subchondral sclerosis and bony irregularity of the posterior talocalcaneal facet of the subtalar joint, leading to the diagnosis of subtalar joint arthritis. An ultrasound-guided intra-articular corticosteroid injection led to rapid pain resolution and a full return to sport within 60 days, with no recurrence at the 90-day follow-up. This case highlights the importance of considering subtalar joint arthritis, particularly involving the posterior talocalcaneal joint, as a differential diagnosis when posterior ankle pain persists despite adequate treatment for os trigonum syndrome. Dynamic ultrasonography is valuable for identifying subtle concomitant pathologies in complex posterior ankle pain.

## Introduction

Ballet movements such as relevé and en pointe involve repetitive extreme plantar flexion, concentrating mechanical stress on the posterior ankle structures [1]. Common etiologies of posterior ankle pain include os trigonum syndrome, flexor hallucis longus (FHL) tendinopathy, and Stieda process impingement [2]. In clinical practice, these conditions may coexist, complicating diagnosis and treatment [2].

We herein present a case of a ballet dancer whose posterior ankle pain was initially attributed to os trigonum syndrome but was ultimately resolved after the diagnosis and treatment of concomitant subtalar joint arthritis through dynamic ultrasonography.

## Case presentation

**Figure 1:**
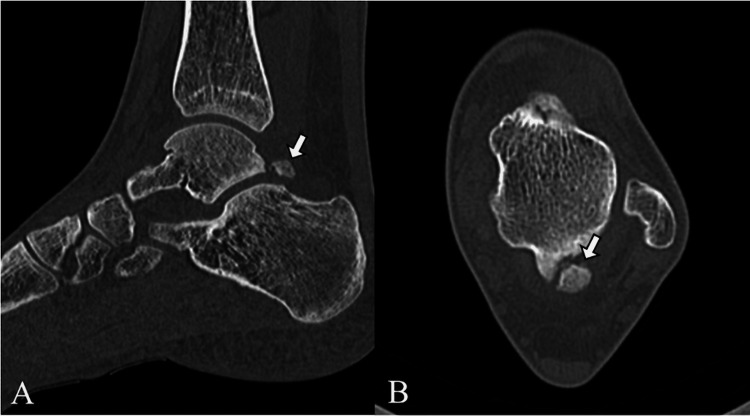
Imaging findings of the os trigonum at initial presentation (computed tomography (CT)) (A) Sagittal view; (B) Axial view. Arrows indicate the os trigonum.

A teenage female ballet student developed left posterior ankle pain during a relevé maneuver. Ten days later, she presented to our clinic. Computed tomography (CT) confirmed the presence of an os trigonum (Figure 1), and ultrasonography revealed a peritendinous Doppler signal around the FHL tendon and hypoechoic changes within the tibiotalar joint. The patient was diagnosed with os trigonum syndrome and advised to refrain from undergoing training for 15 days.

Phase 1: Initial physiotherapy

**Figure 2:**
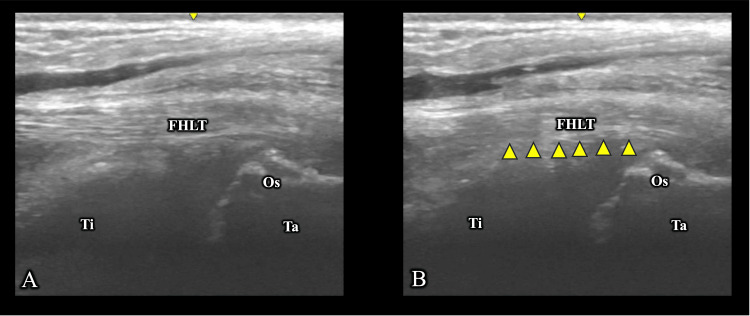
Assessment of flexor hallucis longus (FHL) tendon gliding (A) Resting position; (B) Hallux interphalangeal (IP) joint flexion. FHLT: flexor hallucis longus tendon; Ti: tibia; Os: os trigonum; Ta: talus Arrowheads indicate areas of reduced tendon gliding.

Upon resuming care (day 15), she reported posteromedial pain during relevé (numerical rating scale (NRS) score, 6/10) and limited plantar flexion (85° vs. 90° contralateral). Dynamic ultrasonography confirmed reduced gliding of the FHL tendon (Figure 2). She subsequently underwent two sessions of ultrasound-guided manual therapy, provided once weekly over a two-week period, focusing on enhancing FHL tendon excursion. The pain decreased slightly (NRS 4/10) by day 30; however, functional limitations persisted.

Phase 2 - Re-evaluation

**Figure 3:**
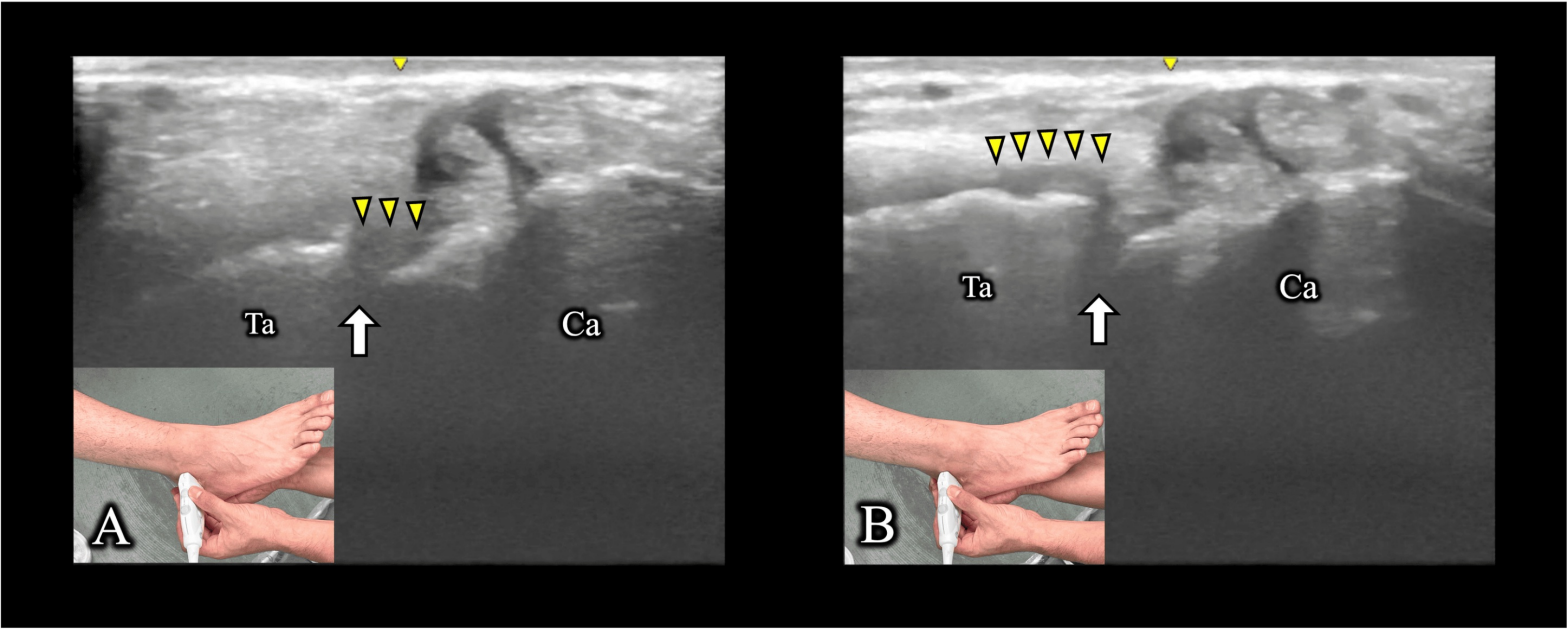
Ultrasound-guided forced pronation maneuver of the subtalar joint in maximal ankle plantar flexion (A) Starting position (maximal ankle plantarflexion, subtalar joint supination); (B) During forced pronation (maximal ankle plantarflexion, subtalar joint pronation). Arrows indicate the subtalar joint; arrowheads indicate hypoechoic areas. Owing to the narrowing of the joint space of the subtalar joint, the hypoechoic area, which is thought to be edema, moved outside the joint. Ta: talus; Ca: calcaneus

**Figure 4:**
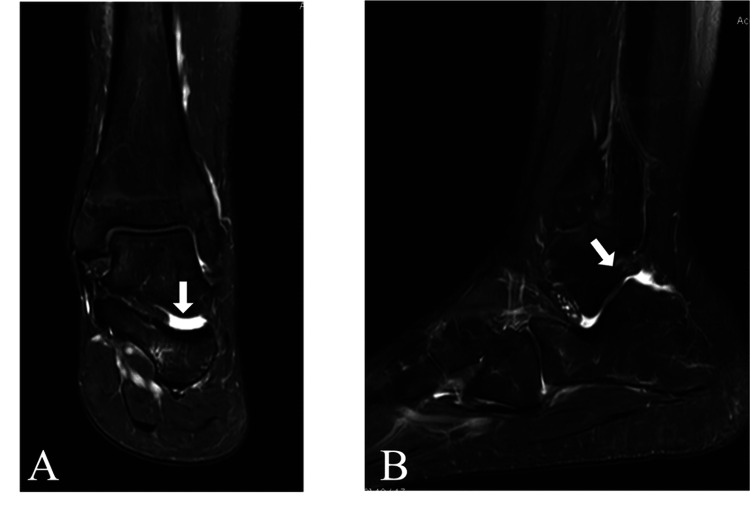
Magnetic resonance imaging (MRI) findings of the subtalar joint (A) Coronal view; (B) Sagittal view. Arrows indicate hyperintense signals suggestive of joint effusion within the subtalar joint.

On day 30, re-evaluation revealed tenderness over the posterior talocalcaneal facet of the subtalar joint. To increase joint stress on the posterior talocalcaneal articular surface, the ankle was placed in maximal plantar flexion with forced subtalar pronation, which reproduced the symptoms [3]. Dynamic ultrasonography demonstrated a hypoechoic area within the posterior talocalcaneal facet of the subtalar joint, which was presumed to be an intra-articular effusion. As the joint space narrowed, the area migrated outside the joint, reproducing the pain (Figure 3). Magnetic resonance imaging demonstrated joint effusion (Figure 4), and CT revealed subchondral sclerosis of the posterior talocalcaneal facet of the subtalar joint.

Ultrasound-guided corticosteroid injection into the posterior talocalcaneal facet of the subtalar joint was performed (Video 1). By day 60, pain had reduced to NRS 1/10, and she returned to full ballet training. At the 90-day follow-up, the patient remained symptom-free. A summary of the clinical course, imaging findings, and interventions is presented in Table 1.

**Video 1:** Ultrasound-guided intra-articular injection into the posterior facet of the subtalar joint

**Table 1:** Summary of the clinical course, imaging findings, and interventions CT: computed tomography; US: ultrasonography; MRI: magnetic resonance imaging; FHL: flexor hallucis longus; NRS: numerical rating scale

Timepoint		Initial visit (10 days after onset)	Day 15 (start of physiotherapy)	Day 30 (re-evaluation)	Day 60	Day 90 (final follow-up)
Clinical findings/Symptoms		Posterior ankle pain during relevé, localized tenderness	Posteromedial pain during relevé, limited plantar flexion (85° vs. 90° contralateral)	Tenderness over the posterior subtalar joint; pain reproduced with maximal plantar flexion + forced pronation	Pain reduction, able to resume ballet training	Symptom-free, full return to ballet
Imaging findings	CT	presence of os trigonum (Figure 1)		Subchondral sclerosis of posterior subtalar joint		
	US	peritendinous Doppler signal around FHL, hypoechoic changes in the tibiotalar joint	reduced FHL tendon gliding (Figure 2)	hypoechoic area in the posterior subtalar joint, consistent with effusion (Figure 3)		
	MRI			joint effusion (Figure 4)		
Treatment / Intervention		Diagnosed with os trigonum syndrome; advised 15-day rest	Ultrasound-guided manual therapy, twice weekly sessions for 2 weeks	Ultrasound-guided corticosteroid injection (Video 1)	Continued physiotherapy	Maintenance therapy
NRS (0–10)		―	6 → 4	4	1	1

## Discussion

Os trigonum syndrome is a well-recognized cause of posterior ankle pain in ballet dancers, with most patients responding to conservative therapy [4]. However, the contribution of subtalar joint arthritis to plantar flexion-related pain has not yet been reported. In the differential diagnosis, although symptoms directly attributable to the os trigonum lesion observed at the initial visit were considered less likely, persistent pain at the same site led us to evaluate the possibility of FHL tendon involvement based on the clinical findings.

In the present case, persistent symptoms after targeted FHL therapy prompted a re-evaluation. Dynamic ultrasonography enabled the visualization of intra-articular changes within the subtalar joint during symptom reproduction, guiding the diagnosis and leading to successful targeted injection therapy. Previous studies have also emphasized that dynamic ultrasonography has diagnostic value in dancers by detecting ankle and foot pathologies that may be overlooked on static imaging [5,6].

Our findings suggest that subtalar joint pathologies should be considered in ballet dancers with unresolved posterior ankle pain following trigonum-directed therapy. Dynamic ultrasonography provides real-time functional assessment that can reveal subtle concomitant lesions that are not evident on static imaging.

## Conclusions

In ballet-specific extreme plantar flexion, posterior ankle pain may result from multiple simultaneous-onset pathologies. This case demonstrates that dynamic ultrasonography is an effective tool for identifying the simultaneous onset of subtalar joint arthritis and os trigonum syndrome, enabling precise intervention and an early return to sport.
